# The correlation between neck circumference and risk factors in patients with hypertension: What matters

**DOI:** 10.1097/MD.0000000000022998

**Published:** 2020-11-20

**Authors:** Yudan Zhang, Haixia Wu, Yilian Xu, Huang Qin, Cuizhen Lan, Wenzhen Wang

**Affiliations:** Department of Nursing, The Second Affiliated Hospital of Hainan Medical University, Hainan Province, China.

**Keywords:** cardiovascular, hypertension, neck circumference, risk

## Abstract

It is necessary to identify the relationship between neck circumference and cardiovascular risk factors in patients with hypertension.

Patients with hypertension treated in our hospital were included. The height, weight, neck circumference, waist circumference, fasting blood glucose, 2 h blood glucose (2hPPG), density lipoprotein cholesterol (LDL-C), high density lipoprotein cholesterol (HDL-C), and glycated hemoglobin (HbA1c) were analyzed and compared.

A total of 2860 patients with hypertension were included. There were significant differences between male and female patients in the neck circumference, waist circumference, fasting blood glucose, Total cholesterol, triacylglycerol, HDL-C, LDL-C, diabetes, metabolic syndrome, dyslipidemia, drinking and smoking (all *P* < .05); the neck circumference was positively correlated with waist circumference, body mass index (BMI), fasting blood glucose, 2hPPG, HbA1c, triacylglycerol and LDL-C (all *P* < .05), and negatively correlated with HDL-C (*P* = .014); as the neck circumference increases, the risk of hypertension, diabetes, metabolic syndrome, abdominal obesity, and dyslipidemia increases accordingly (all *P* < .05); the area under curve (AUC) was 0.827 and 0.812, and the neck circumference of 37.8 and 33.9 cm was the best cut-off point for male and female patients, respectively.

Neck circumference is closely related to cardiovascular risk factors in patients with hypertension, which should be promoted in the screening of cardiovascular diseases.

## 1 Introduction

With the acceleration of the aging process, the elderly population have increased greatly over the past decades. Investigations on the prevalence of chronic diseases in older people is extremely important for targeted prevention and control.^[1]^ Hypertension is an important risk factor for a variety of cardiovascular and cerebrovascular diseases.^[2]^ With increasing age, the prevalence of hypertension increases, and the risk of cardiovascular events also increases significantly. Therefore, understanding the current status of hypertension in the elderly population worldwide is of great significance for grasping the health status of elderly people and providing insights into the healthy policies.

Recent studies^[3,4]^ have reported that different fat distributions can be associated with the hypertension and different metabolic risks. Central obese people, even without significant systemic obesity, may still have metabolic disorders and increase the risk of cardiovascular disease, thereby increasing the morbidity and mortality of hypertension.^[5]^ Central obesity can be measured by different methods, such as neck circumference, waist circumference, waist-hip ratio, and diameter of abdominal dislocation. The neck circumference is not only easy to measure and time-saving, but also it is reported that the neck circumference can not only be used to identify overweight or obese patients, but it may also reflect the type of obesity.^[6,7]^ In recent years studies,^[8,9]^ it has been reported that the in diabetic patients, the neck circumference, as an important indicator of subcutaneous fat in the upper body, is closely related to diabetes and metabolic syndrome, and independently related to the cardiovascular risk factors. At present, there is no large-scale study on the relationship between neck circumference and cardiovascular risk factors in people with hypertension. Therefore, it is necessary to explore the potential relationships between neck circumference and cardiovascular risk factors in patients with hypertension, to identify the role of neck circumference in the development and treatment of hypertension.

## 2 Methods

### 2.1 Ethical considerations

This present study was approved by the Medical Research Ethics Committee of our hospital (No.20180118) and written informed consent was taken from all the patients.

### 2.2 Patients

We selected patients with hypertension who were treated in the Department of Cardiology of our hospital from January 2018 to December 2019 as included participants. The inclusion criteria were: the patient was ≥40 years old, the diagnosis of hypertension met relevant diagnostic criteria, that is, SBP ≥ 140 or DBP ≥ 90 or subject is taking hypertension medication. We excluded patients with thyroid disease, neck tumors, renal insufficiency, or with history of neck surgery.

### 2.3 Data collection

We developed unified form to collect the basic information, health status, diseases history, marriage, and childbearing history, the potential risks such as smoking, drinking, etc. The height, weight, neck circumference, waist circumference, blood pressure were detected and collected from all patients.

We measured the neck circumference as following steps: the patient seat in a sitting position and breathe calmly. The front of the soft ruler was pressed against the lower edge of the thyroid cartilage (larynx node), the back was pressed against the upper edge of the seventh cervical vertebra. We measured the blood pressure as follows: the patient rested for 5 minutes in a sitting position, we measured the blood pressure 3 times with arm blood pressure monitor (Velex 100, Hexi, China) at an interval of 1 minute, we collected the mean blood pressure.

Venous blood was collected from patients on status of empty stomach more than 10 hours to measure fasting blood glucose. Those who had no history of diabetes underwent a 75 g oral glucose tolerance test, those with a history of diabetes underwent a standard steamed bread meal test. Blood was collected again 2 hours after test to determine the 2 hours blood glucose (2hPPG). The remaining specimens were centrifuged on-site and serum was stored at −20 °C and sent to the laboratory department of our hospital for further analysis. Enzymatic methods were used to detect level of total cholesterol and triacylglycerol, and homogeneous methods were used to detect low density lipoprotein cholesterol (LDL-C) and high-density lipoprotein cholesterol (HDL-C). In addition, we used high-performance liquid chromatography to determine level of glycated hemoglobin (HbA1c).

### 2.4 Related diagnostic criteria and definitions

The diagnostic criteria for hypertension referred to the related guidelines,^[10,11]^ which defining hypertension as: systolic blood pressure ≥140 and/or diastolic blood pressure ≥90 mmHg, subject is taking hypertension medication. Dyslipidemia was defined as: total cholesterol ≥5.7 or LDL-C >3.3 or HDL-C <1.0 mmol/L. Abdominal obesity was defined as waist circumference ≥90 cm (male), ≥85 cm (female). The diabetes was defined according to the diagnostic criteria issued by the World Health Organization in 1999: fasting blood glucose ≥7.0 and/or 2hPPG ≥11.1 mmol/L. The diagnosis of metabolic syndrome was based on the related guidelines.^[12,13]^ Smoking was defined as one time per day or at least 7 cigarettes per week. Drinking was defined as at least 1 drink a week for more 6 months.

### 2.5 Statistical analysis

Statistical analysis was performed using SPSS 23.0 software. Measurement data are expressed as mean ± standard deviation (*M* ± SD), group *t* test was used for comparison between 2 groups. Chi-square test was used for comparison of count data. The correlation analyses on neck circumference and related potential cardiovascular risk factors were conducted with Pearson correlation analyses. Logistic regression analyses were conducted to identify the potential risk factors. The receiver operating characteristic (ROC) curve was used to calculate the cutoff value of neck circumference for the diagnosis of the men or women with ≥2 cardiovascular risk factors. In this present study, *P* < .05 was considered statistically significant, all the *P*-values are 2-sided.

## 3 Results

### 3.1 The characteristics of included patients

A total of 2860 patients with hypertension were included in this present study, with 1284 male and 1576 female patients, respectively. As presented in Table 1, there were significant differences between male and female patients in the neck circumference, waist circumference, fasting blood glucose, Total cholesterol, triacylglycerol, HDL-C, LDL-C, diabetes, metabolic syndrome, dyslipidemia, drinking, and smoking (all *P* < .05), while no significant differences in the age, body mass index (BMI), 2hPPG, HbA1c, and abdominal obesity were found (all *P* > .05).

**Table 1:** The characteristics of included patients.

Variables	Male (n = 1284)	Female (n = 1576)	*t*/*χ*^2^	*P*
Age, y	62.91 ± 8.05	63.22 ± 9.19	17.451	.121
Neck circumference, cm	38.95 ± 2.36	34.81 ± 2.40	8.470	.013
Waist circumference, cm	93.32 ± 8.50	88.37 ± 8.28	10.375	.045
BMI, kg/m^2^	25.63 ± 3.08	25.44 ± 3.18	7.285	.308
Fasting blood glucose, mmol/L	5.68 ± 1.34	5.54 ± 1.21	2.583	.047
2hPPG, mmol/L	7.99 ± 1.75	7.98 ± 1.60	1.085	.142
HbA1c (%)	5.94 ± 1.06	5.843 ± 1.13	1.308	.084
Total cholesterol, mmol/L	5.19 ± 1.15	5.69 ± 1.20	1.359	.037
Triacylglycerol, mmol/L	1.44 ± 0.65	1.58 ± 0.72	0.985	.033
HDL-C, mmol/L	1.18 ± 0.63	1.29 ± 0.65	1.145	.046
LDL-C, mmol/L	3.08 ± 1.04	3.22 ± 0.19	1.107	.039
Diabetes (%)	298 (23.21%)	360 (22.84%)	1.316	.044
Metabolic syndrome (%)	688 (53.58%)	747 (47.39%)	2.195	.009
Abdominal obesity (%)	411 (32.01%)	525 (33.31%)	1.108	.124
Dyslipidemia (%)	562 (43.77%)	604 (38.32%)	1.285	.034
Drinking (%)	648 (50.47%)	256 (16.24%)	1.470	.007
Smoking (%)	380 (29.59%)	89 (5.65%)	2.841	.013

2hPPG = 2 h blood glucose, BMI = body mass index, HbA1c = glycated hemoglobin, HDL-C = high density lipoprotein cholesterol, LDL-C = low density lipoprotein cholesterol.

### 3.2 Correlation analysis on neck circumference and related potential cardiovascular risk factors

As presented in Table 2, both for male and female patients with hypertension, the neck circumference was positively correlated with waist circumference, BMI, fasting blood glucose, 2hPPG, HbA1c, triacylglycerol and LDL-C (all *P* < .05), and negatively correlated with HDL-C (*P* = .014).

**Table 2:** Correlation analysis between neck circumference and related potential cardiovascular risk factors.

	Male (n = 1284)		Female (n = 1576)	
Variables	*r*	*P*	*r*	*P*
Waist circumference	0.698	.005	0.648	.016
BMI	0.674	.011	0.620	.009
Fasting blood glucose	0.162	.012	0.017	.015
2hPPG	0.157	.014	0.188	.010
HbA1c	0.142	.031	0.175	.009
Total cholesterol	0.036	.087	−0.091	.255
Triacylglycerol	0.267	.044	0.232	.018
HDL-C	−2.408	.042	−2.357	.014
LDL-C	0.177	.048	0.103	.029

2hPPG = 2 h blood glucose, BMI = body mass index, HbA1c = glycated hemoglobin, HDL-C = high density lipoprotein cholesterol, LDL-C = low density lipoprotein cholesterol.

### 3.3 Logistic regression analysis on the neck circumference and cardiovascular risk factors

As presented in Table 3, both for male and female patients, as the neck circumference increases, the risk of hypertension, diabetes, metabolic syndrome, abdominal obesity, and dyslipidemia increases accordingly (all *P* < .05).

**Table 3:** Logistic regression analysis on the neck circumference and cardiovascular risk factors.

	Dependent variables	*β*	SE	*χ*^2^	OR (95% CI)	*P*
Male (n = 1284)	Diabetes	0.147	0.023	34.680	1.17 (1.10–1.26)	.014
	Metabolic syndrome	0.115	0.026	22.156	1.12 (1.08–1.16)	.008
	Abdominal obesity	0.307	0.028	112.191	1.38 (1.30–1.45)	.015
	Dyslipidemia	0.633	0.047	180.306	1.93 (1.75–2.13)	.006
Female (n = 1576)	Diabetes	0.135	0.017	53.619	1.14 (1.10–1.18)	.014
	Metabolic syndrome	0.156	0.019	69.250	1.16 (1.12–1.20)	.023
	Abdominal obesity	0.191	0.023	194.083	1.37 (1.31–1.42)	.040
	Dyslipidemia	0.085	0.017	4.986	1.03 (0.94–1.12)	.108

### 3.4 The ROC analysis on the neck circumference and cardiovascular disease risks

**Figure 1:**
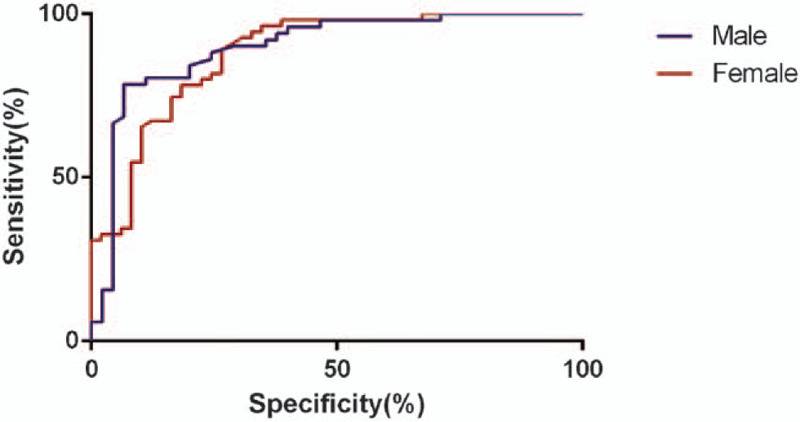
The ROC curve of neck circumference for the diagnosis of the male or female with ≥2 cardiovascular risk factors. ROC = receiver operating characteristic.

The ROC curve analysis was performed using the neck circumference as the detected variable and ≥2 cardiovascular risk factors as the outcome variables. As presented in Fig. 1, the results indicated that for male patients with hypertension, the area under curve (AUC) was 0.827, and the neck circumference of 37.8 cm was the best cut-off point, and the sensitivity and specificity were 82.7% and 68.6%, respectively. For female patients with hypertension, the AUC was 0.812, and the neck circumference of 33.9 cm was the best cut-off point, and the sensitivity and specificity were 80.5% and 66.3%, respectively.

## 4 Discussion

Hypertension, as an important risk factor for cardiovascular and cerebrovascular diseases such as coronary heart disease and stroke, has become a global health problem. Recent studies^[14–16]^ have shown that overweight and obese people have a 2 to 3-fold increased risk of developing hypertension compared with people with normal weight. Besides, many studies^[17,18]^ have reported that the neck circumference as an index to assess the subcutaneous fat of the upper body is also related to hypertension, but the results are not consistent. The results of this present study have indicated that with the increase of neck circumference, the risk of hypertension, diabetes, metabolic syndrome, abdominal obesity, and dyslipidemia will increase accordingly, neck circumference is a good indicator with high sensitivity and specificity for hypertension.

Waist circumference, BMI, and waist-to-hip ratio are the traditional indicators for obesity diagnosis, which have been confirmed by numerous studies^[19–21]^ to be related to cardiovascular events. Neck circumference is also a window of body fat distribution, it is more stable and simple to measure neck circumference than waist circumference, especially in the screening process, neck circumference can be measured in a sitting position without interference from breathing.^[22,23]^ In recent years, many epidemiological studies^[24–26]^ have suggested that neck circumference is closely related to waist circumference and BMI, thus it is an important indicator for predicting risk factors for cardiovascular disease. Furthermore, neck circumference is closely related to glucose and lipid metabolism disorders, insulin resistance, etc, and is related to various components of metabolic syndrome.^[27,28]^ Previous study^[29]^ has included 3037 Caucasians aged 40 to 60 years old, the results have suggested that neck circumference is associated with blood pressure, blood glucose, lipids, and insulin level. A study^[30]^ with 4201 Chinese included by has pointed out that the neck circumference was related to the triacylglycerol and fasting blood glucose, which is consistent with our findings.

Neck circumference is an important indicator on the distribution of subcutaneous fat in the upper body.^[31]^ Subcutaneous fat in the upper body has been proven to be an important source of free fatty acid release in the circulatory system, and especially in obese people.^[32,33]^ The increase in free fatty acid concentration can lead to increased release of inflammatory factors, which can result in insulin resistance and vascular endothelial damage.^[34]^ Furthermore, neck circumference has good predictive value for the risk of sleep apnea syndrome,^[35,36]^ while sleep apnea syndrome is also closely related to a variety of cardiovascular diseases.

The mechanism of neck circumference and hypertension is not yet clear, it may be explained from following aspects. Firstly, if the subcutaneous fat represented by the neck circumference increases, it leads to a significant increase in F2-isoprostaglandin levels, thus causing vascular endothelial injury.^[37]^ Secondly, increased neck circumference promotes the upper body subcutaneous adipose tissue, in which mature adipocytes continuously synthesize and secrete adipokines such as leptin to increase the excitability of sympathetic nerves, and release catecholamines to cause arterial contraction.^[38]^ Thirdly, those patients with increased neck circumference generally have decreased insulin sensitivity, leading to insulin resistance and compensatory hyperinsulinemia.^[39,40]^ Selective impairment of the insulin signaling pathway of vascular endothelial cells reduces the production and release of nitric oxide, which leads to vasodilation dysfunction and arterial stiffness, which promotes the occurrence of hypertension.^[41]^ Fourthly, those with increased neck circumference often have more subcutaneous fat, which may release more free fatty acids, increase the production of oxygen free radicals and enhance oxidative stress response, which lead to the vascular endothelial dysfunction, and finally causing the blood pressure to rise.^[42,43]^ Therefore, tracking changes of neck circumference is beneficial to understand the status of cardiovascular system.

There are several shortcomings in this present study. First, the study sample in this study came from one hospital, the area represented by the population was very limited, and the major limitation of the ROC analysis is that the discovered neck circumference cutoffs are purely data driven, based on the authors’ own data. There is no guarantee that these cutoffs will apply to the population in general. Thus related studies on different population are needed in the future. Second, limited by the sample size, we could not perform further sub-group analysis stratified by the severity of hypertension. Future analysis would be better served by a linear regression analysis showing the beta coefficients (per 1 cm or 1 standard deviation increase in neck circumference) and their 95% confidence intervals. This way, we can assess not only the strength of the association, but also the magnitude of the association, future analyses on this are needed. Third, we only performed single measurement of the neck circumference, multiple repeated measurements for precision may be more appropriate in the future study. The correlation of hypertension severity with the comorbidities should be considered, for example, the association may be higher in those patients with grade 2 of hypertension, future studies focused on this issue are needed.

## 5 Conclusions

In conclusion, the results of this study have showed that in patients with hypertension, neck circumference is independently associated with the cardiovascular risk factors. When the neck circumference is ≥37.8 cm for men and ≥33.9 cm for women, it indicates that there may be ≥2 risk factors for cardiovascular disease. Neck circumference has good predictive effect on early detection of cardiovascular risk factors in people, which is worthy of being promoted in the screening process of physical examinations. However, limited by the small size, future studies with larger sample size and different areas are warranted to identify the role of neck circumference in cardiovascular diseases.

## Author contributions

**Conceptualization:** Yudan Zhang, Haixia Wu, Yilian Xu, Cuizhen Lan.

**Data curation:** Haixia Wu, Yilian Xu, Wenzhen Wang.

**Formal analysis:** Yudan Zhang.

**Funding acquisition:** Yudan Zhang, Yilian Xu.

**Investigation:** Yilian Xu.

**Methodology:** Haixia Wu, Yilian Xu, Huang Qin.

**Project administration:** Yilian Xu, Wenzhen Wang.

**Resources:** Yilian Xu.

**Software:** Haixia Wu, Yilian Xu, Wenzhen Wang.

**Supervision:** Yilian Xu, Huang Qin.

**Validation:** Cuizhen Lan.

**Visualization:** Yilian Xu.

**Writing – original draft:** Yilian Xu.
